# Mid- and long-term clinical outcomes of corrective fusion surgery which did not achieve sufficient pelvic incidence minus lumbar lordosis value for adult spinal deformity

**DOI:** 10.1186/1748-7161-10-S2-S17

**Published:** 2015-02-11

**Authors:** Kentaro Yamada, Yuichiro Abe, Yasushi Yanagibashi, Takahiko Hyakumachi, Shigenobu Satoh

**Affiliations:** 1 Department of Orthopaedic Surgery, Wajokai Eniwa Hospital, 2-1-1 Koganechuo, Eniwa, Hokkaido 061-1449, Japan

## Abstract

**Background:**

Recent studies have demonstrated sagittal spinal balance was more important than coronal balance in terms of clinical result of surgery for adult spinal deformity. Notably, Schwab reported that one of the target spinopelvic parameters for corrective surgery was that pelvic incidence (PI) minus lumbar lordosis (LL) should be within +/- 10 °. The present study aimed to investigate whether the clinical outcome of corrective fusion surgery was really poor for patients who could not acquire sufficient PI-LL value through the surgery.

**Methods:**

The present study included 13 patients (mean 68.5 yrs old) with adult spinal deformity. Inclusion criteria were corrective fusion surgery more than 4 intervertebral levels, PI-LL ≥10° on the whole spine X-ray immediately after surgery, and follow-up period ≥3 years. All surgeries were performed by posterior approach. Parameters using SRS-Schwab classification, proximal junctional kyphosis (PJK) of ≥15°, implants loosening, and non-union were investigated using the total standing spinal X-ray. Clinical outcomes were evaluated by Japanese Orthopaedic Association scores (JOA score), Oswestry Disability Index, SF-36, Visual Analog Scale for low back pain, and satisfaction for surgery using SRS-22 questionnaire.

**Results:**

All patients showed the PI-LL ≥20° before surgery. Although the LL were acquired mean 23.6° after surgery, significant loss of correction was observed at final follow up. The acquired coronal spinal alignment was maintained within the follow-up period. However, sagittal vertical axis (SVA) was shifted forward significantly, from mean 4.5cm immediately after surgery to 11.1cm at final follow-up. Five patients showed PJK, 10 patients showed implants loosening, 8 patients showed non-union at final follow-up. The JOA score and mental health summary measures of SF-36 were significantly improved at final follow-up. The satisfaction score was mean 3.3 points, including 3 patients with ≥4 points, at final follow-up. The satisfaction score correlated negatively with SVA at final follow-up (ρ=-0.58 p=0.03).

**Conclusions:**

The forward shift of SVA was frequently observed, and SVA at final follow-up related to the patient’s satisfaction of surgery. This study indicated the importance of postoperative PI-LL value, but also noted 23% of patients acquired good SVA and satisfaction nevertheless they had inadequate postoperative LL.

## Background

The prevalence of adult spinal deformity among elderly people has been reported as high as 60% [1]. Although the majority of cases with deformity are asymptomatic, the others have pain, neural symptoms, functional limitation, or disability. Non-operative treatment should be attempted initially for those patients. Operative treatment of spinal corrective surgery may be considered for in cases with severe pain or disability despite sufficient conservative therapy. Sagittal spinal balance was reportedly more important than coronal balance in terms of clinical result of corrective fusion surgery in recent years [2,3]. Notably, Schwab reported one of the target spinopelvic parameters for corrective surgery was that pelvic incidence (PI) minus lumbar lordosis (LL) should be within +/- 10 ° [3]. However, little is known about whether clinical outcomes are poor in all cases which could not achieve the target of PI-LL value. The present study was aimed to investigate clinical outcomes and individual satisfaction for surgery in patients who could not acquire sufficient PI-LL value after corrective fusion surgery.

## Methods

The present study investigated patients with adult spinal deformity who underwent spinal corrective fusion surgery more than 4 intervertebral levels. Inclusion criteria of the adult spinal deformity were preliminary degenerative scoliosis [4] or degenerative kyphosis over 50 yrs ord. Patients with progressive idiopathic scoliosis, kyphosis due to vertebral fractures or Scheuermann’s disease were excluded. Between 1999 and 2010, a total of 31 patients underwent spinal corrective fusion surgery more than 4 intervertebral levels, for adult spinal deformity at our institution. All patients had severe low back pain and functional disability with and without neurological symptom despite sufficient conservative therapy. The present study included patients who showed PI-LL ≥10° immediately after surgery, and were followed up ≥3 years. A total of 13 patients (2 males, 11 females) met inclusion criteria. The age at the time of surgery was 55 to 82 years (mean, 68.5 yr). The follow-up period was 3 to 10 years (mean, 5.1 yr). All surgeries were performed by posterior approach. The number of fusion levels was 4 to 8 levels (mean 6.0 levels). Posterior lumbar interbody fusion was performed with 0 to 4 levels (mean 2.8 levels). Pedicle-subtraction-osteotomies were combined in 5 patients.

Radiological evaluations were investigated by standing whole spineX-ray. Radiological parameters using SRS-Schwab classification [5]; Cobb angle, LL (L1-S1), Sacral vertical axis (SVA: C7 plumb line relative to S1), pelvic tilt (PT), and PI-LL, were investigated preoperative, immediately after surgery, and at final follow-up. Proximal junctional kyphosis (PJK: sagittal Cobb angle between the upper instrumented vertebra [UIV] and the vertebra 2 levels above the UIV) of ≥15°, implants loosening, and non-union were evaluated at final follow-up. Clinical outcomes were evaluated by Japanese Orthopaedic Association scores (JOA score), Oswestry Disability Index (ODI), SF-36, Visual Analog Scale (VAS) for low back pain, and satisfaction for surgery using SRS-22 questionnaire. The study was approved by the Ethical Committee for Clinical Research at Wajokai Eniwa Hosiptal (approval date: April 17, 2013).

Mean and standard deviation were used to describe continuous variables. Changes in radiological and clinical parameters in each time point were evaluated by using a paired t-test analysis. Correlative relationships were analyzed using Spearman’s correlation analysis. A p value of ≤0.05 was considered statistically significant.

## Results

**Figure 1:**
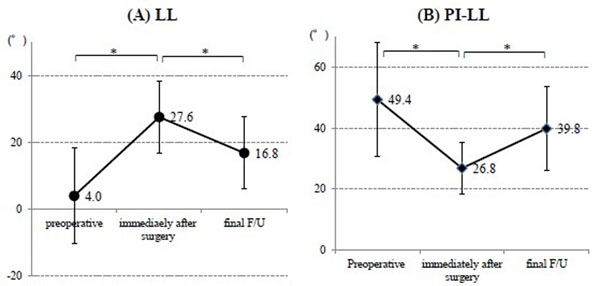
**Changes of (A) lumbar lordosis and (B) Pelvic incidence minus lumbar lordosis.** (A) Lumbar lordosis (LL) showed significant improvement after surgery, but significant loss of correction at final follow-up (F/U). (B) pelvic incidence (PI) – LL also showed same as the movement of LL. * means p<0.05.

**Figure 2:**
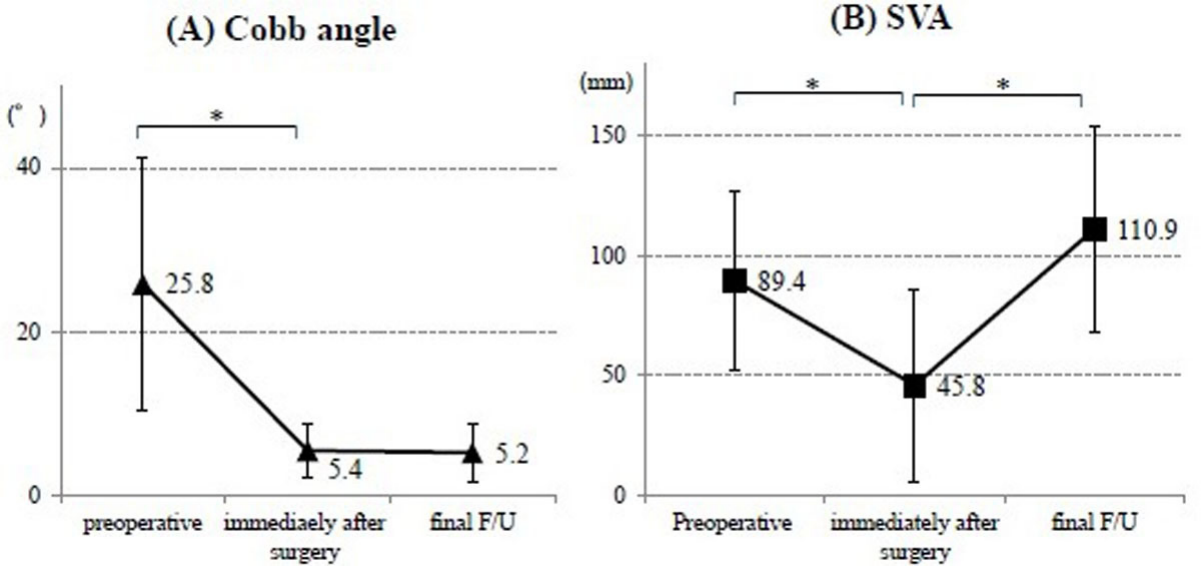
**Changes of (A) Cobb angle and (B) Sagittal vertical axis.** (A) Coronal Cobb angle was improved significantly after surgery and maintained at final follow-up (F/U). (B) Sagittal vertical axis (SVA) showed significant improvement after surgery, but showed significant anterior shift at final F/U. * means p<0.05.

**Figure 3:**
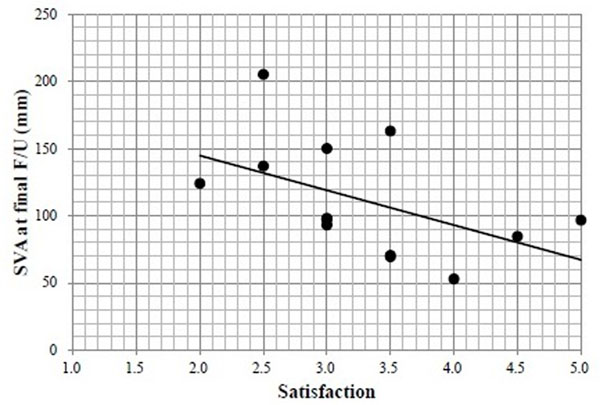
**Relationship between satisfaction and sagittal vertical axis.** Satisfaction correlated negatively with sagittal vertical axis (SVA) at final follow-up (F/U) (ρ=-0.58 p=0.03). There were 3 patients (23%) who satisfied for surgery (≥4points).

All patients showed the PI-LL ≥20° before surgery. There were 5 patients with preoperative coronal Cobb angle ≥30° and 5 patients with preoperative SVA ≥9.5cm. Although the LL was acquired 23.6±16.1° after surgery, significant loss of correction (10.8±5.7°) was observed at final follow up (Figure 1A). PI-LL also showed significant improvement by surgery, but showed significant loss of correction at final follow up (Figure 1B). The acquired coronal spinal alignment was maintained within the follow-up period (Figure 2A). However, SVA was significantly shifted forward from 4.5±4.0cm immediately after surgery to 11.1±0.4cm at final follow-up (Figure 2B). PJK was showed in 5 patients, implants loosening in 10 patients, and non-union in 8 patients at final follow-up. The JOA score and Mental Component summary of SF-36 were significantly improved at final follow-up. The other clinical outcomes were improved but not significant (Table 1). The satisfaction score was 3.3±0.8 points, including 3 patients with ≥4 points, at final follow-up. The satisfaction score correlated negatively with SVA at final follow-up (Figure 3).

**Table 1:** Clinical outcomes after surgery

	Preoperative	Final F/U	p
JOA score	14.6±5.8	21.0±5.0	<0.01
ODI	37.6±12.5	32.5±22.8	0.93
SF36 PCS	25.9±10.1	36.0±13.2	0.14
MCS	40.8±12.1	54.1±9.8	<0.01
VAS (LBP)	57.4±31.0	36.2±28.9	0.08
VAS (leg pain)	49.9±10.9	41.8±34.8	0.98
VAS (leg numbness)	45.2±40.9	26.8±30.0	0.35

JOA, Japanese Orthopaedic Association; ODI, Oswestry Disability Index (ODI); PCS, Physical Component summary; MCS, Mental Component summary; VAS, Visual Analog Scale; LBP, low back pain

## Discussion

Schwab proposed that realignment objectives were SVA less than 5cm, PT less than 25°, and LL proportional to the PI [3]. Among these parameters, LL is the most changeable parameter by surgery, in contrast to PI which is a fixed morphologic parameter in each person [6]. PI-LL mismatch has been reported as key radiographic parameters associated deterioration of health-related quality of life (HRQOL) both in patients under conservative and operative treatment [7-10]. The present study investigated clinical outcomes in patients exhibited PI-LL mismatch after surgery in the era before the proposal by Schwab. The radiological and clinical outcomes were not excellent on the whole. In particular, SVA shifted forward ≥2cm at final follow-up compared with that immediately after surgery in 11 of 13 patients, and SVA at final follow-up was associated with patient’s satisfaction with the surgery. This might have arisen from PI-LL mismatch at the surgery, consistent with previous studies. However, there were 3 cases (23%) that showed good results for both sagittal balance and satisfaction. Therefore, it could still be controversial whether all these cases needed more highly invasive surgery in order to acquire more lordosis, although the small sample size is the major limitation of this study.

Weakness or atrophy of the lumbar extensor muscles was often observed in patients who showed lumbar degenerative kyphosis in past reports [11]. Although this study did not include factors pertaining to back muscle, the strength of back muscle might affect postoperative deterioration of sagittal balance. Future studies should be performed including the evaluation of back muscle, and investigate its effects to the postoperative sagittal balance.

## Conclusions

The forward shift of SVA was frequently observed, and SVA at final follow-up related patient’s satisfaction of surgery. This study indicated the importance of postoperative PI-LL value, but also noted 23% of patients acquired good SVA and satisfaction nevertheless they had inadequate postoperative LL.

This is the extended abstract of IRSSD 2014 program book [12].

## Consent to participate

Written informed consent was obtained from the patient in this study. A copy of the written consent is available for review by the Editor of this journal.

## Conflict of interests

The authors declare that they have no competing interests.

## Authors' contributions

The clinical study was designed by KY, YA and SS, and performed by KY, YA, YY, TK, and SS. Data analyzed by KY. The article is written by KY and YA.

## Declarations

Publication charges for this collection were funded by IRSSD 2014 Sapporo.

This article has been published as part of *Scoliosis* Volume 10 Supplement 2, 2015: Research into Spinal Deformities 9: Short Papers from the IRSSD 2014 Meeting. The full contents of the supplement are available online at http://www.scoliosisjournal.com/supplements/10/S2.
